# Increased Insulin Secretion and Glucose Effectiveness in Obese Patients with Type 2 Diabetes following Bariatric Surgery

**DOI:** 10.1155/2023/7127426

**Published:** 2023-11-14

**Authors:** Roberto Visentin, Katrine Brodersen, Bjørn Richelsen, Niels Møller, Chiara Dalla Man, Andreas Kristian Pedersen, Jan Abrahamsen, Jens Juul Holst, Michael Festersen Nielsen

**Affiliations:** ^1^Department of Information Engineering, University of Padova, Padova, Italy; ^2^Department of Surgery, Viborg General Hospital, Denmark; ^3^Steno Diabetes Center Aarhus, Aarhus University Hospital & Clinical Medicine, Aarhus University, Denmark; ^4^Department of Clinical Research, University Hospital of Southern Denmark, Denmark; ^5^Department of Radiology, Viborg General Hospital, Denmark; ^6^Novo Nordisk Foundation, Center of Basic Metabolic Research and Department of Biomedical Sciences, The Panum Institute, University of Copenhagen, Denmark; ^7^Department of Surgery, University Hospital of Southern Denmark, Denmark

## Abstract

**Background:**

*β*-cell dysfunction and insulin resistance are the main mechanisms causing glucose intolerance in type 2 diabetes (T2D). Bariatric surgeries, i.e., sleeve gastrectomy (SG) and Roux-en-Y gastric bypass (RYGB), are procedures both known to induce weight loss, increase insulin action, and enhance *β*-cell function, but hepatic insulin extraction and glucose effectiveness may also play a role.

**Methods:**

To determine the contribution of these regulators on glucose tolerance after bariatric surgery, an oral glucose tolerance test (OGTT) was performed before and 2 months after surgery in 9 RYGB and 7 SG subjects. Eight healthy subjects served as metabolic controls. Plasma glucose, insulin, C-peptide, GLP-1, and GIP were measured during each OGTT. Insulin sensitivity and secretion, glucose effectiveness, and glucose rate of appearance were determined via oral minimal models.

**Results:**

RYGB and SG resulted in similar weight reductions (13%, RYGB (*p* < 0.01); 14%, SG (*p* < 0.05)). Two months after surgery, insulin secretion (*p* < 0.05) and glucose effectiveness both improved equally in the two groups (11%, RYGB (*p* < 0.01); 8%, SG (*p* > 0.05)), whereas insulin sensitivity remained virtually unaltered. Bariatric surgery resulted in a comparable increase in the GLP-1 response during the OGTT, whereas GIP concentrations remained unaltered. Following surgery, oral glucose intake resulted in a comparable increase in hepatic insulin extraction, the response in both RYGB and SG patients significantly exceeding the response observed in the control subjects.

**Conclusions:**

These results demonstrate that the early improvement in glucose tolerance in obese T2D after RYGB and SG surgeries is attributable mainly to increased insulin secretion and glucose effectiveness, while insulin sensitivity seems to play only a minor role. This trial is registered with NCT02713555.

## 1. Introduction

Glucose intolerance in obese type 2 diabetics (T2D) is determined by defects in insulin secretion and action. Bariatric surgery conducted either as a Roux-en-Y gastric bypass procedure (RYGB) or a sleeve gastrectomy (SG) is the most effective treatment for achieving not only sustained and significant weight loss but also significant metabolic improvements that go beyond the effects of mere weight reduction. Furthermore, bariatric surgery induces remission and has been demonstrated to prevent and delay the onset of T2D [1, 2]. The metabolic interactions causing these improvements seem to be multifactorial, involving genetic, metabolic, psychological, and neuroendocrine mechanisms [3, 4]. Although these mechanisms for the most part are well described, it remains largely unresolved whether the responses differ between RYGB and SG patients.

It is well described that the improvement in glucose tolerance takes place shortly after surgery and occurs even before a significant weight loss can be demonstrated [2, 5]. A consistent observation seems to be that weight loss is greater following RYGB than after SG and that T2D remission is approximately 10% higher after RYGB than after SG. Among those patients experiencing T2D remission, the T2D relapse is lower after RYGB than after SG. Furthermore, HbA1_C_ is lower 5 years after RYGB than after SG. These observations have been confirmed in a recent review by Borgeraas et al. [1], in which the remission rate of T2D at 1 year is described as higher among patients undergoing RYGB (57%) than after SG (47%).

Furthermore, a variety of studies demonstrate that incretin hormones play a critical role in the improvement of glucose homoeostasis. Studies conducted by Færch et al. suggest that a reduction in the GLP-1 response occurs before the development of T2D [6], and experiments by Jørgensen et al. have demonstrated that excess in GLP-1 is essential for the improvement in glucose tolerance after RYGB [7]. While these trials strongly suggest that GLP-1 is pivotal for obtaining enhanced glucose tolerance after RYGB surgery, it remains unresolved whether this response differs in SG patients.

Due to the difference in upper GI anatomy, splanchnic glucose uptake is likely to differ in RYGB and SG patients. This difference may alter the profile for postprandial glucose appearance and affect the glycemic response following oral glucose intake.

The change in the glycemic response induced by the alterations in glucose production is likely to be present shortly after the operation because the improvement in glycemic control in diabetic patients can be observed even before a significant weight loss has occurred. While the long-term effects on glucose tolerance appear to be linked to the improvement in insulin action, the short-term effects are more complex and likely affected by the combined interactions of neurohormonal mechanisms regulating glucose production. This effect has been observed in both clamp studies [8–10] and studies examining the effect of bariatric surgery on the expression and regulation of proteins involved in the regulation of peripheral glucose metabolism [11]. While these studies suggest that improved peripheral insulin action is important for obtaining long-term remission of T2D, insulin action appears to be less important for the improvement in glucose tolerance immediately after surgery. The latter is controversial because the short-term effects of bariatric surgery have not been thoroughly investigated, likely because studies examining the immediate effects of bariatric surgery on glucose metabolism have to be conducted in the presence of physiologic, i.e., variable glucose and insulin concentrations, where glucose kinetics due to non-steady-state conditions is complex and difficult to determine. The present study is aimed at examining these mechanisms by using the classic oral minimal model to calculate postprandial indices of glucose metabolism and compare these in SG and RYGB patients 2 months after surgery [12–19]. This methodological approach is novel and has to our knowledge not previously been used to examine these effects in SG and RYGB patients.

## 2. Materials and Methods

### 2.1. Participants

After approval from the local Ethical Review Board, County of Aarhus, obese T2D subjects referred for bariatric surgery and healthy nondiabetic subjects gave written consent to participate in the study (NCT02713555). Twenty-two individuals preparing for bariatric surgery from the waiting list at the Departments of Endocrinology, Aarhus University Hospital, and Viborg Regional Hospital were assessed for the studies. Two subjects were excluded due to high glucose levels. Three subjects withdrew from operation, and one subject did not complete the study and was therefore excluded from the analysis.

The indication for bariatric surgery was BMI > 35 kg/m^2^. Only subjects fulfilling the requirements for T2D were included in the study. T2D was defined as HbA1_C_ above 48 mmol/mol or treated with antidiabetic agents for T2D. Prior to surgery, subjects had to demonstrate a weight loss of at least 8% of total body weight. The type of surgery was decided in collaboration between the subject and the endocrinologist. Altogether, 16 obese individuals with T2D, 9 RYGB and 7 SG, and 8 nondiabetic control subjects with a BMI of 25 kg/m^2^ were recruited for the studies. The characteristics of the study subjects are shown in Table 1.

Diabetes remission at 2 months of follow-up was defined as HbA1_C_ less than 48 mmol/mol without antidiabetic medication or HbA1_C_ less than 42 mmol/mol with metformin therapy.

### 2.2. Experimental Design

Healthy control subjects underwent a single oral glucose tolerance test (OGTT) on the day of the study. The obese T2D subjects underwent two OGTTs, one before the bariatric surgery and one two months after the intervention. Three days prior to study, subjects were instructed not to engage in vigorous exercise and to remain on the diet recommended by the Department of Endocrinology, Aarhus University Hospital. Following an overnight fast, subjects were admitted to the General Medical Research Center, Aarhus University Hospital. An 18-gauge catheter was inserted into a forearm vein and used for all infusions. A second cannula was inserted retrogradely into a dorsal hand vein and placed in a heated Plexiglas box. The temperature was maintained at 55°C to allow sampling of arterialized venous blood.

### 2.3. Oral Glucose Tolerance Test

Baseline samples for glucose, insulin, and C-peptide were obtained at -30, -15, and 0 min before the OGTT. The oral glucose tolerance test (OGTT) consisted of 50 g glucose ingested at time 0 as detailed in [20]. The amount of glucose intake was selected by the fact that the volume of the residual stomach is significantly reduced after bariatric surgery, especially following RYGB. On the contrary, a standard 75 g OGTT would require a large volume, likely making the study subjects vomit following the glucose intake. Additionally, bariatric patients are prone to develop dumping symptoms following a large carbohydrate intake. Thus, reducing the glucose load to 50 g was necessary for all study subjects to complete the study.

Blood samples were drawn for measurement of glucose, insulin, and C-peptide at time *t* = −120, -30, -15, 0, 5, 10, 15, 20, 30, 45, 60, 75, 90, 120, 150, 180, and 240 min, while incretin hormones (GLP-1, GIP) were measured at *t* = –120, -15, 0, 15, 30, 45, 60, 90, 120, 180, and 240 min.

### 2.4. Analytical Techniques

Body composition and lean body mass were measured by dual-energy X-ray absorptiometry (DXA). Arterialized plasma samples were placed on ice, centrifuged at 4°C, separated, and stored at -20°C until assay. Blood samples were collected in tubes containing 50 mmol/L EDTA plus 500 kallikrein inhibitory units/ml aprotinin for measurement of GLP-1, GIP, and C-peptide. All samples were extracted in a final concentration of 70% ethanol before GLP-1 and GIP analyses. Total GLP-1 was measured as previously described [21] using a radioimmunoassay (antibody code no. 89390) specific for the C-terminal part of the GLP-1 molecule and reacting equally with intact GLP-1 and the primary (N-terminally truncated) metabolite. Total GIP concentration was measured with a radioimmunoassay using an antibody directed towards the C-terminus (code no. 80867), which reacts fully with intact GIP and N-terminally truncated forms [22]. Sensitivity for both assays was below 1 pmol/L and intra-assay coefficient of variation below 10%. Glucose concentrations were measured using a Yellow Springs glucose analyzer (Yellow Springs Instruments, Yellow Springs, OH). Plasma insulin and C-peptide concentrations were measured by enzyme-linked immunosorbent assay kits (ELISA) (Mercodia, Sweden). This assay was 100% specific fort GLP-1 and does not cross-react with glucagon, GLP-2, or ghrelin.

### 2.5. Oral Minimal Models

Key parameters of the glucose regulatory system were assessed using the oral minimal models. These models, previously validated against both clamp and intravenous glucose tolerance tests [23–26], account for a few numbers of parameters that can be estimated from the data, thus enabling to describe and evaluate certain variables and mechanisms not directly measurable.

In particular, the oral glucose minimal model [27] describes plasma glucose dynamics from plasma insulin concentration and administered exogenous glucose. It provides estimates of insulin sensitivity (*S*_I_), an index representing the insulin-driven suppression of endogenous glucose production and promotion of glucose disposal, glucose effectiveness (GE), and exogenous glucose rate of appearance (Ra). In order to account for the potential change in meal glucose absorption after gastric surgery, we calculated the area under the curve (AUC) of Ra in the first 60 min after glucose ingestion, normalized by the total orally absorbed glucose (AUC(Ra_0-60_)).

The insulin and C-peptide minimal model [28] describes the plasma insulin and C-peptide concentrations in relation to the observed changes in glucose concentration and provides estimates of basal and total hepatic insulin extraction (HE_b_ and HE_tot_, respectively) and insulin and C-peptide secretion by means of *β*-cell responsivity indices, i.e., dynamic (*Φ*_d_), static (*Φ*_s_), basal (*Φ*_b_), and total (*Φ*_tot_) responses to the glycemic stimulus. *β*-cell function can be further expressed in light of the prevailing *S*_I_ through the disposition indices (DI_d_, DI_s_, DI_tot_) introduced in [29, 30] defined as (*Φ*_d_, *Φ*_s_, *Φ*_tot_) × *S*_I_. Further information on model equations and calculation of indices is provided in [31].

### 2.6. Statistical Analysis

Variables are reported as median [25^th^, 75^th^] percentile for each outcome, unless otherwise stated. Two-way analysis of variance and Bonferroni's test were used to assess differences between treatment groups (RYGB vs. SG vs. control) and visits (pre- vs. postoperative). A *p* value < 0.05 was considered statistically significant. The post hoc analysis was performed using Student's *t*-test for normally distributed variables, or Wilcoxon's test otherwise, and within each treatment group by paired test, or by unpaired test among treatment groups. For each treatment group, the outcome deviation (%) was calculated as the difference between postoperative and preoperative values, i.e., (outcome_post_–outcome_pre_)/outcome_pre_. Correlations between *β*-cell responsivity indices and incretin hormones were assessed by calculating Pearson's linear correlation coefficient for normally distributed outcomes, or Spearman's rho otherwise.

## 3. Results

### 3.1. Body Mass Index and Total and Lean Body Weight

The effects of the bariatric procedures on the anthropometric parameters are outlined in Table 1. BMI showed a median reduction by 13% (*p* < 0.001) and 14% (*p* = 0.016) in RYGB and SG subjects, respectively; TBW was reduced by 13% (*p* < 0.001) and 14% (*p* < 0.001), and LBM decreased by 8% (*p* < 0.001) and 9% (*p* = 0.014). However, all parameters were still significantly higher (*p* < 0.001) than the corresponding measured in the healthy control subjects.

### 3.2. Plasma Glucose, Insulin, and C-Peptide Concentrations during OGTT

**Figure 1:**
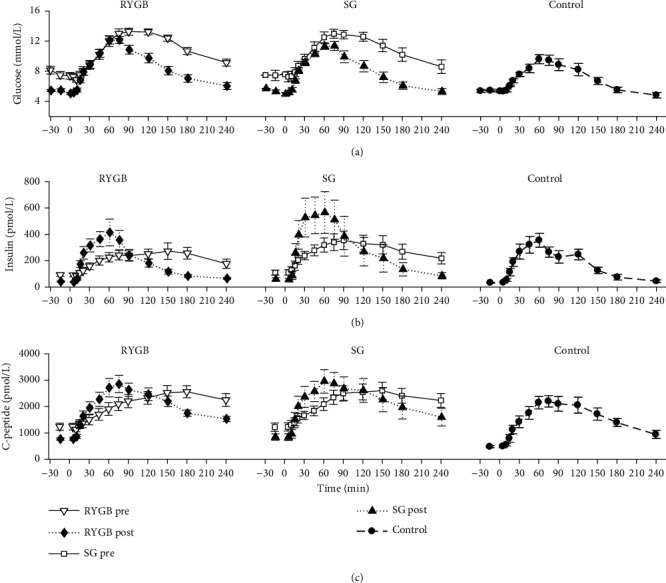
Average ± standard error (SE) time courses of plasma glucose (a), insulin (b), and C-peptide (c) measured before and 2 months after gastric bypass (RYGB, *left*) or sleeve gastrectomy (SG, *center*), or in the healthy control subjects (*right*).

The average fasting plasma glucose, insulin, and C-peptide concentrations (*C*_0_) are reported in Table 1 for pre- and postoperative visits and groups. Postprandial time course of plasma glucose, insulin, and C-peptide concentrations in the bariatric subjects, before and after RYGB and SG, and in the control subjects is shown in Figure 1. After surgery, glucose area under the curve (AUC) significantly decreased from 24.2 [23.5, 25.7] to 19.0 [17.9, 22.0] 10^2^ mmol·min/L (*p* = 0.017) in the RYGB group and from 23.2 [22.0, 26.9] to 17.3 [16.7, 19.3] 10^2^ mmol·min/L (*p* = 0.006) in the SG group; the reduced AUC after surgery was similar to that measured in the control group (15.9 [15.1, 17.8] 10^2^ mmol·min/L). Glucose peak values were not significantly different from pre- (13.5 [13.0, 15.1] and 14.4 [12.2, 14.9] mmol/L) to postoperative visit (13.1 [12.3, 13.3] and 11.1 [11.0, 11.7] mmol/L), remaining significantly higher than the control group (9.8 [8.7, 11.1] mmol/L, *p* = 0.002 and *p* = 0.049, respectively). However, time-to-peak was reduced after surgery (from 90.0 [71.3, 120.0] to 60.0 [45.0, 60.0] min and from 60.0 [60.0, 82.5] to 45.0 [45.0, 60.0], in RYGB and SG groups, respectively), and similar to that of the control group (45.0 [45.0, 60.0] min).

With respect to plasma insulin and C-peptide, there were no significant differences in AUC, either within (i.e., before vs. after surgery) or between groups. However, after surgery, insulin peak was significantly increased in both the RYGB (from 309.0 [183.0, 408.0] to 424.0 [290.3, 653.5] pmol/L, *p* = 0.008) and the SG group (from 351.0 [287.5, 554.8] to 497.0 [341.3, 1140.8] pmol/L, *p* = 0.037).

### 3.3. Insulin Action and Secretion

#### 3.3.1. Insulin Action

**Figure 2:**
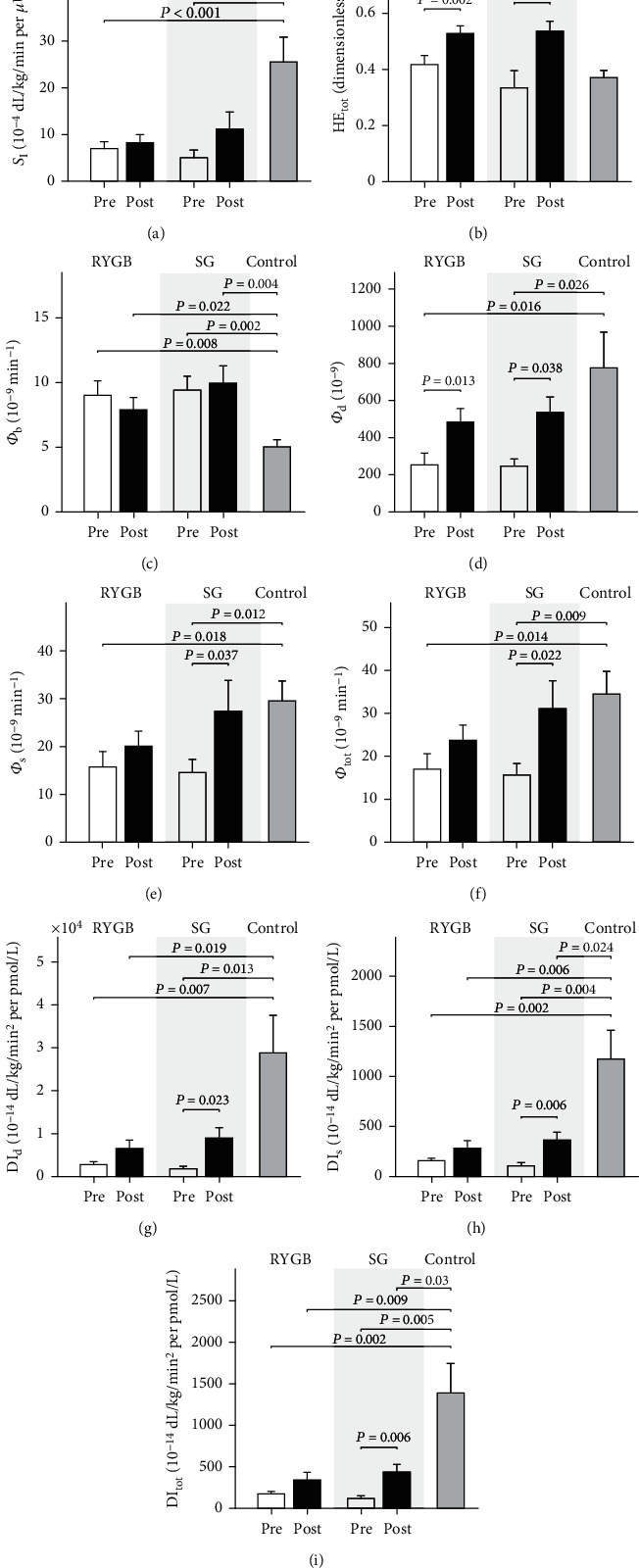
Mean ± standard error (SE) of insulin sensitivity (*S*_I_, a); total hepatic extraction (HE_tot_, b); basal (*Φ*_b_, c), dynamic (*Φ*_d_, d), static (*Φ*_s_, e), and total *β*-cell responsivity index (*Φ*_tot_, f); and dynamic (DI_d_, g), static (DI_s_, h), and total disposition index (DI_tot_, i) estimated preoperative (white bars) and 2 months postoperative (black bars), or in the healthy control subjects (*textured bar*). Bars are grouped by surgery group, i.e., gastric bypass (RYGB, *left*), sleeve gastrectomy (SG, *center*), and control (*right*).

Compared to the preoperative estimates, *S*_I_ was slightly but not significantly increased following RYGB and SG with a median increase of 26% (*p* = 0.442) and 11% (*p* = 0.524), respectively. Differences in insulin action between the two groups were not observed, neither before nor two months after the surgical procedures. Compared to healthy control subjects, *S*_I_ remained impaired in both bariatric groups (*p* = 0.005 and *p* = 0.048 in RYGB and SG, respectively) (Figure 2(a) and Table 2).

#### 3.3.2. Hepatic Insulin Extraction

Both RYGB and SG resulted in an increase in HE_b_ (median increase by 35% and 36%, respectively) and HE_tot_ (median increase by 31% and 48%, respectively), both exceeding those observed in the healthy control subjects (Figure 2(b) and Table 2). The increase did not differ between the two bariatric groups.

#### 3.3.3. Insulin Secretion

Compared to the control group, *Φ*_b_ was increased preoperatively in the bariatric groups and remained unaltered after RYGB and SG (Figure 2(c) and Table 2). *Φ*_d_ was impaired prior to surgery and increased equally after RYGB and SG by 132% (*p* = 0.013) and 173% (*p* = 0.038), respectively, to a rate comparable to that observed in the control subjects (Figure 2(d) and Table 2). *Φ*_s_ and *Φ*_tot_ were also both impaired prior to surgery, but in contrast to the RYGB, where *Φ*_s_ and *Φ*_tot_ remained statistically unaltered, they increased after SG by 82% (*p* = 0.037) and 90% (*p* = 0.022), respectively (Figures 2(e) and 2(f) and Table 2). As observed for *Φ*_d_, the postoperative increase in both *Φ*_s_ and *Φ*_tot_ was comparable to that of the healthy control subjects.

#### 3.3.4. Disposition Index

SG resulted in an increase in all the disposition indices, with a median improvement of 285% (*p* = 0.023), 178% (*p* = 0.006), and 209% (*p* = 0.002) for DI_d_, DI_s_, and DI_tot_, respectively. Similarly, RYGB demonstrated a trend towards an increase of these indices; however, this rise was not insignificant due to larger variability. Postoperatively, all disposition indices in the bariatric subjects remained lower than those observed in the control subjects (Figures 2(g)–2(i) and Table 2).

### 3.4. Glucose Effectiveness

RYGB resulted in a significantly increased GE (*p* = 0.010) with a median improvement of 11%, while in the SG group, the change (8%) was not significant. Compared to healthy control, GE remained impaired following both RYGB (*p* < 0.001) and SG (*p* = 0.002) (Table 2).

### 3.5. Exogenous Glucose Absorption

**Figure 3:**
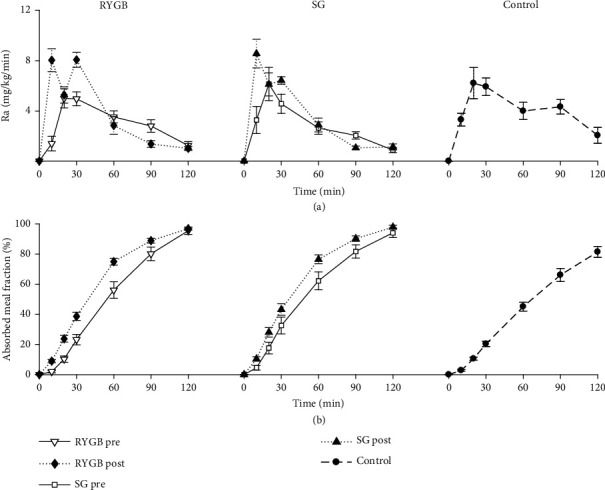
Mean ± standard error (SE) time courses of meal glucose rate of appearance (a) and percent absorbed meal fraction between 0 and 60 min (b) estimated before and 2 months after gastric bypass (RYGB, *left*) or sleeve gastrectomy (SG, *center*), or in the healthy control subjects (*right*).

The rate of external glucose appearance increased at 2 months after surgery (Figure 3(a)). In particular, AUC(Ra_0-60_) was significantly higher following RYGB, with a median increase of 36% (*p* = 0.007). Compared to the control group, AUC(Ra_0-60_) was higher following both RYGB and SG (Figure 3(b)).

### 3.6. Incretin Hormones and Insulin Secretion

**Figure 4:**
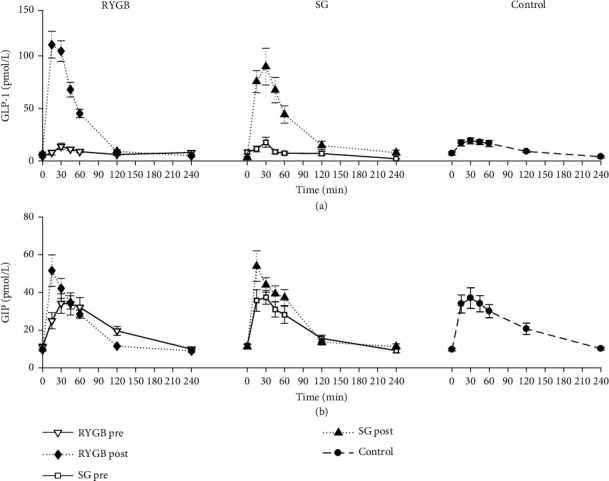
Mean ± standard error (SE) time courses of GLP-1 (a) and GIP (b) measured before and 2 months after gastric bypass (RYGB, *left*) or sleeve gastrectomy (SG, *center*), or in the healthy control subjects (*right*).

Incretin concentrations time courses are shown in Figure 4. Postabsorptive GLP-1 concentrations did not differ at *t* = 0 between obese T2D (4.0 [2.0, 4.5] pmol/L and 8.0 [5.0, 12.0] in RYGB and SG, respectively) and the healthy control subjects (7.0 [4.0, 11.0] pmol/L, *p* = 0.160) and remained unaltered in all groups during the OGTT. Postabsorptive GIP concentrations did not differ between T2D (10.0 [8.5, 13.3] pmol/L and 12.0 [11.0, 13.0] in RYGB and SG, respectively) and control subjects (9.0 [8.5, 10.5] pmol/L, *p* = 0.500), and during the OGTT, GIP concentrations increased comparably before and after surgery in all groups. Following surgery, oral glucose ingestion resulted in a 10-fold increase in GLP-1 concentrations in both the RYGB and SG subjects with no difference in response between the two groups. The distribution of GIP time-to-peak appeared anticipated after surgery, especially for the RYGB group. However, these differences were not statistically significant, neither within (i.e., before vs. after surgery) nor between groups.

**Figure 5:**
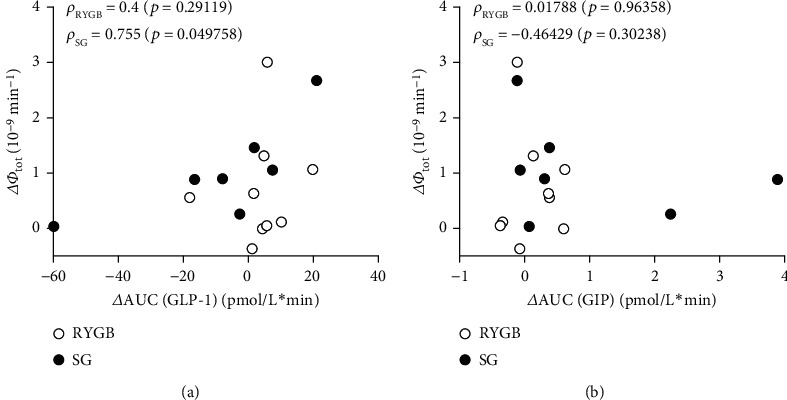
Correlation between the incremental increase in total insulin secretion index (Δ*Φ*_tot_) and incremental increase in area under GLP-1 (*Δ*AUC(GLP-1), a) and GIP (*Δ*AUC(GIP), b) concentrations. RYGB: Roux-en Y gastric bypass; SG: sleeve gastrectomy.

Whereas GLP-1 responses during the OGTT were similar in the control and the diabetic subjects, RYGB and SG resulted in a comparable increase in GLP-1 that significantly exceeded the response observed in the controls. In contrast, GIP increased comparably during the OGTT in the control and the T2D subjects, and this response did not differ before and after surgery. In order to investigate possible relationships between the increase in insulin secretion and the rise in incretin levels, the incremental insulin responses were correlated to the corresponding increments in GLP-1 and GIP concentrations. As depicted in Figure 5, a borderline significant correlation (*p* = 0.049) with change in total *β*-cell responsivity to glucose (Δ*Φ*_tot_) could be established for GLP-1 in the SG group, whereas no correlation could be established for GIP.

## 4. Discussion

Bariatric surgery induces weight loss and improves glycemic control, but despite a large number of studies, the mechanisms leading to improved glucose tolerance in T2D remain complex and require further investigation. In particular, the contribution of insulin secretion and action, glucose effectiveness, and hepatic insulin extraction to improve glucose tolerance immediately after bariatric surgery and whether these indices differ between RYGB and SG patients remain largely unknown. To address this question, an oral minimal model analysis was performed based on an OGTT conducted before and 2 months after RYGB and SG surgeries in obese individuals with T2D. The metabolic effects of the RYGB and SG procedures were assessed by calculating indices for insulin secretion and action, hepatic extraction, and glucose effectiveness and comparing these indices between the two bariatric groups.

Our study demonstrates a significant but comparable improvement in glucose tolerance 2 months after RYGB and SG surgeries, a response which was demonstrated to be due mainly to an increase in insulin secretion and glucose effectiveness. Noteworthy, insulin sensitivity remained virtually unaltered. These results imply that while insulin secretion and glucose effectiveness are responsible for the immediate improvement in glucose tolerance, enhanced insulin action appears to be a long-term effect likely associated to the extent of weight loss.

Similarly, glucose effectiveness and hepatic insulin extraction were both comparably increased in the two groups. These observations suggest that insulin secretion and glucose effectiveness and likely also hepatic insulin extraction are major determinants for the immediate improvement in glucose tolerance after bariatric surgery and that these changes may occur independent of an increase in insulin sensitivity.

To the best of our knowledge, these observations have not previously been reported. While the improvement in glucose tolerance after RYGB and SG surgeries has been demonstrated in the previous studies [32–35], the present study is the first to investigate the short-term effects on glucose tolerance using the minimal model analysis for OGTT data [31]. This model allows the estimation of insulin sensitivity, beta-cell responsivity indices, hepatic insulin extraction, and glucose effectiveness following oral glucose intake, thus taking into account the effects of variable glucose and hormone concentrations on key estimates of glucose metabolism, which is not possible using the glucose clamp or IVGTT study design. As such, this study indirectly provides a validation of the previous findings [8–10].

A significant observation is that the postoperative rise in insulin secretion was observed coincident with a rise in the disposition index (DI). Since DI is calculated as the product between insulin secretion and insulin sensitivity, the increase in the DI index induced by both RYGB and SG provides further evidence to support that insulin secretion is a major factor responsible for the improvement in glucose tolerance after bariatric surgery.

In contrast to the present study, results reported by Fatima et al. [36] demonstrate that RYGB is associated with a greater improvement in *β*-cell function and a higher postprandial GLP-1 response than that observed after SG surgery. These results differ from the observations reported in the present study, where no difference in the GLP-1 response could be demonstrated. Based solely on the data at hand, it is difficult to speculate on the reason for this discrepancy. A plausible explanation could be a type 2 error, which would imply that a potential difference between the two groups in the present study has been overlooked. Irrespective of the cause, the results from the two studies consistently demonstrate an increase in insulin secretion following bariatric surgery, and the data thus provide strong evidence to suggest that insulin secretion is a major determinant for the improvement in glucose tolerance after both SG and RYGB surgeries.

The present study does not directly determine the underlying causes leading to enhanced insulin secretion and hyperinsulinemia, but it seems likely that the response is linked to the regulation of the enteroinsular axis, which has been demonstrated to be sensitive to changes in nutrients presented to the intestine [37]. Both SG and RYGB result in altered transit time of nutrients through the upper GI tract, and it seems likely that an altered carbohydrate load presented to the mucosa cells of the small intestine is responsible for the increase in insulin secretion observed after the bariatric procedures [38–40]. Concentration of the insulinotropic hormones, in particular, GLP-1, which is released in the distal ileum and colon, has been found to be increased after gastric bypass surgery in several studies [41, 42]. In the present study, we observed a comparable increase in GLP-1 in both the RYGB and SG subjects. In contrast, GIP remained unaltered and did not differ between the two bariatric groups. In addition, a weak correlation was established between GLP-1 and the insulin responsivity index, whereas this could not be demonstrated for GIP. This implies that GLP-1 is likely to play a key role for the regulation of insulin secretion, whereas GIP does not. However, these correlations also suggest that other currently not identified factors are likely to play a role for the postprandial rise in insulin secretion [43]. In addition to GLP-1, the enteroinsular axis comprises numerous and probably also unknown hormones that influence glucose metabolism and these hormones may therefore contribute to the altered insulin response observed in the present study.

Whereas hepatic insulin extraction was equally increased in both groups following surgery, it appears noteworthy that the response in both groups exceeded that observed in the healthy individuals. Hepatic insulin clearance is believed to be mediated by receptor binding and has been proposed to be coupled to hepatic insulin sensitivity [44]. Short-term caloric restriction in patients with T2D improves hepatic insulin sensitivity independently of weight loss [45, 46] and is associated with reduction in liver fat content [47, 48]. A similar mechanism may be present after bariatric surgery [49]. Insulin clearance is a saturable process, which needs to be considered in patients with high portal insulin concentrations [44]. In the present study, hepatic insulin extraction was increased following both RYGB and SG, which cannot be explained by receptor saturation alone. In contrast, the data may imply an increase in insulin binding to hepatocytes as previous studies suggest that RYGB induces early changes in hepatic rather than peripheral insulin action [50]. Another mechanism may be related to the rise in insulin secretion. Hepatic insulin clearance has been demonstrated to respond rapidly to dynamic changes in insulin secretion, and the rise in hepatic insulin extraction may occur as a result of this phenomenon [51, 52].

Glucose effectiveness, i.e., the ability of glucose *per se* to suppress glucose production and stimulate glucose uptake, has been demonstrated to be a significant factor for the regulation of glucose homeostasis in both healthy subjects and in T2D [53–55]. It has been estimated that in normal individuals, approximately 50% of glucose disposal during an OGTT is due to glucose effectiveness and not to the dynamic insulin response [56], and several studies have demonstrated glucose effectiveness to be impaired in T2D [57–59]. In insulin-resistant obese individuals, more than 80% of glucose disposal occurs independently of the dynamic insulin response, which is likely to be increased in individuals with T2D with severe insulin resistance and relative insulinopenia. Thus, glucose effectiveness appears to be a factor at least equal to insulin itself in the determination of glucose tolerance [56], highlighting the finding in the present study of an increase of 11% and 8% of glucose effectiveness in the RYGB and SG subjects, respectively.

It is worth noting that the differences observed between the two groups might be caused uniquely by the surgical procedure *per se* or also a consequence of the small sample size. Indeed, this latter aspect represents an important limitation of the present study, together with a gender unbalance between RYGB and SG groups of the study. In this study, the type of surgical procedure was not randomly assigned, as it was decided based on the patient's clinical status, so that the perfect distribution of woman and men among study groups was difficult to grant. Gender effect on metabolic indices has been evaluated in a previous study [12], resulting in statistically significantly higher glucose effectiveness in women than men, lower insulin action in young (i.e., <30 years) women than young men, and no differences in insulin secretion and hepatic extraction. Based on this information, glucose effectiveness might have been overestimated in the RYGB study group (women only), while insulin action might have been underestimated. Therefore, future analyses in larger and gender equally distributed populations will be required to unveil the complex nature of the mechanisms causing enhanced glucose tolerance after bariatric surgery. In addition, despite the primary aim of this study being to assess the short-term effects of RYGB and SG, it would be interesting to perform longitudinal studies to determine the long-term effects of bariatric surgery on glucose tolerance.

Another limitation is that the model analysis required for making assumptions rests on few parameter values. The impact of such assumptions has been widely discussed in previous publications [23, 24, 30, 31]. Future experiments using the oral minimal model with dual glucose tracers would allow the estimation of both hepatic and disposal components of insulin sensitivity [60]. This could reveal possible significant improvement in one of the two contributions, as in the present work, an increasing trend in net insulin action (despite not statistically significant) was observed.

In conclusion, the present study provides significant new insights about glucose tolerance restoration after bariatric surgery in obese subjects with T2D showing that RYGB and SG have acute effects in increasing insulin secretion, hepatic insulin extraction, and, to a lesser extent, glucose effectiveness.

## Figures and Tables

**Table 1:** Metabolic response rate distributions.

	RYGB	SG	Control
#	9	7	8
Age (years)	49 [47, 53]	49 [47, 51]	49 [43, 61]
Sex (female)	9	3	4
Preoperative			
BMI (kg/m^2^)	41.9 [41.8, 45.5]^§^	40.5 [39.6, 44.8]^§^	25.4 [24.6, 26.2]
TBW (kg)	120.5 [117.2, 127.6]^§^	129.6 [111.8, 143.2]^§^	73.7 [69.4, 78.1]
LBM (kg)	64.4 [57.4, 65.7]^§^	79.3 [57.4, 84.9]^§^	48.9 [41.0, 57.9]
Glucose			
*C*_0_ (mmol/L)	7.1 [6.7, 8.2]^§^	6.5 [6.2, 7.8]^§^	5.3 [4.9, 5.7]
*C*_max_ (mmol/L)	13.5 [13.0, 15.1]^§^	14.4 [12.2, 14.9]^§^	9.8 [8.7, 11.1]
*t*_max_ (min)	90.0 [71.3, 120.0]^§^	60.0 [60.0, 82.5]	45.0 [45.0, 60.0]
AUC (mmol/L·min)	24.2 [23.5, 25.7]^§^	23.2 [22.0, 26.9]^§^	15.9 [15.1, 17.8]
Insulin			
*C*_0_ (mmol/L)	74.0 [65.6, 107.5]^§^	117.5 [76.3, 155.5]^§^	33.5 [28.5, 37.8]
*C*_max_ (pmol/L)	309.0 [183.0, 408.0]	351.0 [287.5, 554.8]	377.0 [274.5, 489.0]
*t*_max_ (min)	90.0 [56.3, 150.0]	60.0 [37.5, 108.8]	45.0 [30.0, 67.5]
AUC (pmol/L·min)	5.1 [3.1, 6.6]	5.3 [4.9, 8.1]	3.8 [2.5, 5.0]
C-peptide			
*C*_0_ (mmol/L)	1091.5 [1011.0, 1468.5]^§^	1229.5 [953.8, 1602.8]^§^	480.8 [417.3, 517.0]
*C*_max_ (pmol/L)	2606.0 [1835.0, 3506.3]	2515.0 [2216.3, 3043.8]	2271.0 [2047.0, 2775.0]
*t*_max_ (min)	120.0 [112.5, 150.0]^§^	120.0 [75.0, 120.0]^§^	67.5 [52.5, 90.0]
AUC (pmol/L·min)	5396.0 [3726.2, 6360.9]	4871.4 [4651.2, 5901.8]	4079.4 [3368.6, 4528.8]
Postoperative			
BMI (kg/m^2^)	38.1 [35.7, 39.5]^∗^^§^	34.6 [33.4, 38.2]^∗^^§^	
TBW (kg)	107.5 [99.0, 111.7]^∗^^§^	109.9 [102.4, 122.5]^∗^^§^	
LBM (kg)	59.2 [52.5, 60.9]^∗^^§^	70.0 [57.6, 77.1]^∗^^§^	
Glucose			
*C*_0_ (mmol/L)	5.0 [4.9, 5.6]^∗^	5.3 [4.7, 5.5]^∗^	
*C*_max_ (mmol/L)	13.1 [12.3, 13.3]^§^	11.1 [11.0, 11.7]^§^	
*t*_max_ (min)	60.0 [45.0, 60.0]^∗^	45.0 [45.0, 60.0]	
AUC (mmol/L·min)	19.0 [17.9, 22.0]^∗^	17.3 [16.7, 19.3]^∗^	
Insulin			
*C*_0_ (mmol/L)	36.0 [28.1, 52.5]^∗^	57.5 [38.8, 83.1]^∗^^§^	
*C*_max_ (pmol/L)	424.0 [290.3, 653.5]^∗^	497.0 [341.3, 1140.8]^∗^	
*t*_max_ (min)	45.0 [30.0, 60.0]^∗^	30.0 [20.0, 56.3]	
AUC (pmol/L·min)	3.3 [2.8, 4.7]	4.1 [3.2, 9.2]	
C-peptide			
*C*_0_ (mmol/L)	703.5 [674.0, 864.5]^∗^^§^	968.5 [585.3, 1064.0]^∗^^§^	
*C*_max_ (pmol/L)	3378.0 [1962.3, 3714.5]	2785.0 [2408.5, 4148.0]	
*t*_max_ (min)	60.0 [45.0, 63.8]^∗^	45.0 [33.8, 56.3]^∗^^§^	
AUC (pmol/L·min)	4919.3 [3530.5, 5382.5]	4501.7 [3881.3, 6727.9]	

Values are reported as median [25^th^, 75^th^] percentile range. ^∗^Significant *p* < 0.05 compared to preoperative. ^§^Significant *p* < 0.05 compared to healthy control. RYGB: Roux-en Y gastric bypass; SG: sleeve gastrectomy.

**Table 2:** Minimal model outcome distributions.

Index	RYGB	SG	Control
S_I_ (10^−4^ dL/kg/min per *μ*U/mL)			
Preoperative/control	5.0 [4.7, 7.5]^§^	4.4 [1.5, 8.7]^§^	22.3 [13.8, 38.7]
Postoperative	8.9 [4.1, 12.0]^§^	8.5 [5.0, 15.3]^§^	
HE_b_ (dimensionless)			
Preoperative/control	0.48 [0.43, 0.55]	0.44 [0.38, 0.50]	0.51 [0.47, 0.58]
Postoperative	0.61 [0.59, 0.69]^∗^^§^	0.60 [0.60, 0.64]^∗^^§^	
HE_tot_ (dimensionless)			
Preoperative/control	0.40 [0.37, 0.48]	0.39 [0.19, 0.40]	0.39 [0.32, 0.41]
Postoperative	0.54 [0.51, 0.59]^∗^^§^	0.50 [0.47, 0.58]^∗^^§^	
*Φ*_b_ (10^−9^ min^−1^)			
Preoperative/control	9.0 [6.9, 11.5]^§^	9.8 [9.2, 10.9]^§^	5.3 [4.2, 5.6]
Postoperative	7.2 [6.0, 11.2]^§^	10.6 [8.4, 12.8]^§^	
*Φ*_d_ (10^−9^)			
Preoperative/control	208.8 [112.8, 379.5]^§^	259.9 [205.6, 313.1]^§^	621.1 [391.1, 1250.5]
Postoperative	495.3 [330.7, 596.5]^∗^	633.0 [376.9, 733.5]^∗^	
*Φ*_s_ (10^−9^ min^−1^)			
Preoperative/control	11.6 [9.2, 20.9]^§^	13.6 [8.2, 19.2]^§^	29.4 [20.7, 39.7]
Postoperative	19.8 [11.9, 25.5]	21.5 [12.8, 43.6]^∗^	
*Φ*_tot_ (10^−9^ min^−1^)			
Preoperative/control	13.3 [9.5, 22.1]^§^	14.8 [9.4, 20.4]^§^	33.2 [23.7, 49.3]
Postoperative	26.1 [14.2, 30.0]	26.7 [16.4, 48.5]^∗^	
DI_d_ (10^−14^ dL/kg/min per pmol/L)			
Preoperative/control	2887.5 [876.9, 3437.3]^§^	1461.4 [444.2, 3230.8]^§^	18349.3 [11958.0, 42693.5]
Postoperative	6087.3 [1737.7, 8284.3]^§^	9482.9 [2791.6, 13799.9]^∗^	
DI_s_ (10^−14^ dL/kg/min^2^ per pmol/L)			
Preoperative/control	174.4 [80.8, 208.5]^§^	131.3 [34.9, 147.4]^§^	1037.4 [624.6, 1432.2]
Postoperative	255.4 [65.7, 426.7]^§^	386.6 [296.2, 427.1]^∗^^§^	
DI_tot_ (10^−14^ dL/kg/min^2^ per pmol/L)			
Preoperative/control	197.2 [83.2, 234.5]^§^	133.7 [38.5, 159.7]^§^	1172.2 [711.9, 1658.1]
Postoperative	293.5 [75.9, 487.8]^§^	419.7 [350.4, 531.2]^∗^^§^	
GE (dL/kg/min)			
Preoperative/control	0.041 [0.039, 0.045]^§^	0.032 [0.029, 0.044]^§^	0.059 [0.054, 0.066]
Postoperative	0.046 [0.044, 0.051]^∗^^§^	0.047 [0.040, 0.050]^§^	
AUC(Ra_0-60_) (%)			
Preoperative/control	84 [76, 112]^§^	95 [77, 115]^§^	67 [53, 72]
Postoperative	114 [108, 133]^∗^^§^	112 [109, 121]^§^	

Values are reported as median [25^th^, 75^th^] percentile range. ^∗^Significant *p* < 0.05 compared to preoperative. ^§^Significant *p* < 0.05 compared to healthy control. RYGB: Roux-en Y gastric bypass; SG: sleeve gastrectomy.

## Data Availability

Original data generated and analyzed during this study are included in this published article or in the data repositories listed in the references.

## Abbreviations

SG: Sleeve gastrectomy
LBM: Lean body mass
OGTT: Oral glucose tolerance test
RYGB: Roux-en-Y gastric bypass
TBW: Total body weight
T2D: Type 2 diabetes.
